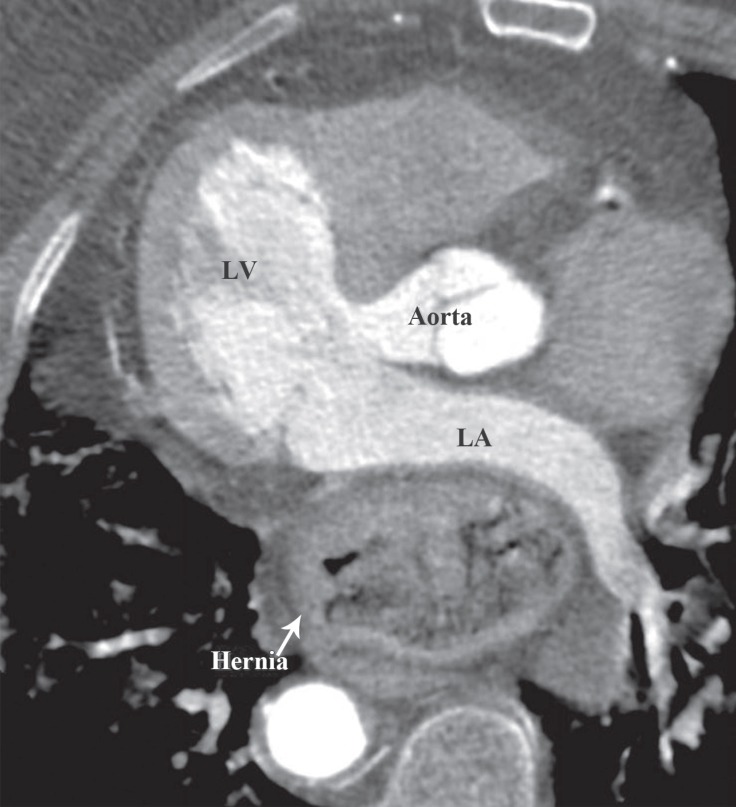# Huge Hiatal Hernia Mimicking a Mass with Compressive Effects on the Left Atrium Causing Paroxysmal Atrial Fibrillation

**Published:** 2019-04

**Authors:** Murat Akçay, İlkay Çamlıdağ

**Affiliations:** 1 *Department of Cardiology, Faculty of Medicine, Ondokuz Mayıs University, Samsun, Turkey. *; 2 *Department of Radiology, Faculty of Medicine, Ondokuz Mayıs University, Samsun, Turkey.*

**Keywords:** *Hernia, hiatal*, *Heart atria*, *Atrial fibrillation*

An 82-year-old female patient presented with complaints of dyspnea and increasing palpitations caused by food reflux. There was no risk factor except hypertension. On physical examination, the heart rate was 120 beats/min and arrhythmic and blood pressure was 130/80 mmHg. Electrocardiography showed high-rate atrial fibrillation. Laboratory parameters were unremarkable. Echocardiography illustrated a hyperechogenic and well-circumscribed mass, 40×55 mm size, in the posterior left atrium (Figure 1, Video 1). The mass size increased with breathing and the Valsalva maneuver. There was no pathology on chest radiography. The atrial fibrillation returned to sinus rhythm spontaneously, but paroxysmal atrial fibrillation attacks were observed, which were related to food reflux at follow-up. Subsequently, cardiac computed tomography, performed to determine the etiology, failed to demonstrate any pathological findings involving the left atrium. However, there was a sliding hernia in the paraesophageal region compressing the left atrium from the inferior-posterior region (Figure 2). Hiatal hernia surgery was recommended on account of the intermittently repeating symptoms. The patient refused the operation, and she is under follow-up with medical treatment. 

**Figure:**
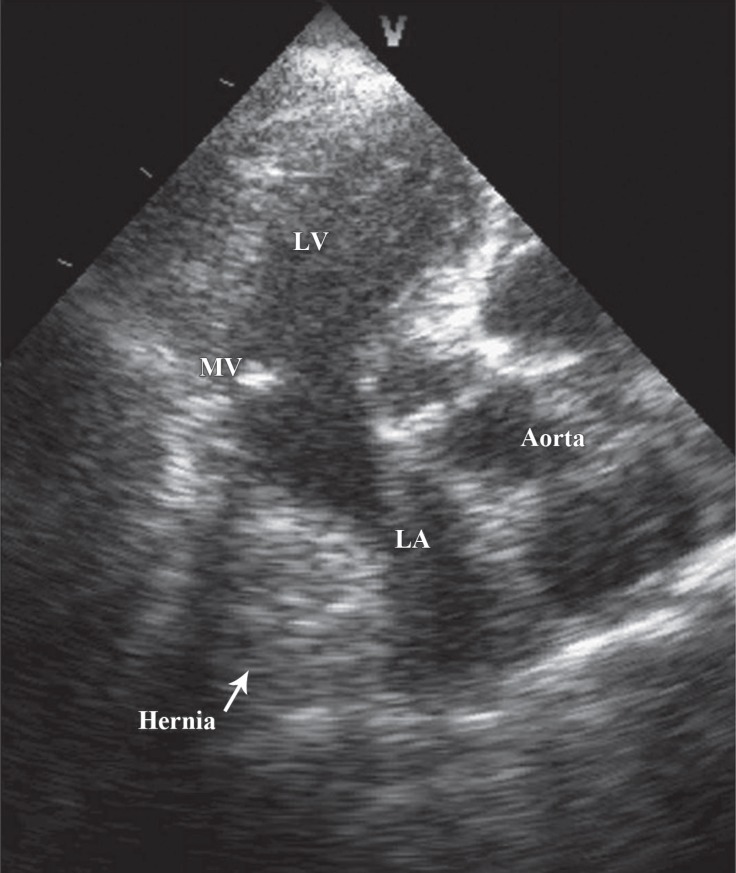


Hiatal hernias are described as abnormal protrusions of the stomach through the diaphragmatic esophageal hiatus. They are usually latent, with symptoms often related to gastroesophageal reﬂux signs. Huge hernias can rarely be misdiagnosed as intracardiac masses during echocardiography. Additionally, they can cause paroxysmal atrial fibrillation attacks due to symptomatic left atrial compression and the irritation of the vagus nerve. Huge hiatal hernias may mimic cardiac masses and rarely may cause paroxysmal atrial fibrillation attacks, as was the case in our patient. They should, be carefully differentiated from other cardiac pathologies.

**Figure:**